# High‐Definition tDCS of the DLPFC: Effects on Effort‐Related Cardiac Reactivity Across Sexes

**DOI:** 10.1111/psyp.70214

**Published:** 2026-01-11

**Authors:** David Framorando, Andréa Razzetto

**Affiliations:** ^1^ Geneva Motivation Lab, FPSE, Section of Psychology University of Geneva Geneva Switzerland; ^2^ Swiss Center for Affective Sciences University of Geneva Geneva Switzerland

**Keywords:** approach/avoidance motivation, effort, pre‐ejection period, transcranial direct current stimulation (tDCS)

## Abstract

This study was designed to examine the effect of frontal hemispheric asymmetry (FHA) on effort‐related cardiovascular responses. High‐definition transcranial direct current stimulation (HD‐tDCS) was applied to the dorsolateral prefrontal cortex (dlPFC) to manipulate FHA and investigate its impact on cardiovascular reactivity. The sample consisted of 45 female and 44 male participants, who received either left or right cathodal stimulation. Following stimulation, participants performed two types of task demands: one fixed and easy, the other unfixed. In both tasks, participants could earn a moderate monetary reward. We measured pre‐ejection period (PEP), heart rate (HR), systolic blood pressure (SBP), and diastolic blood pressure (DBP). Drawing on motivation intensity theory (MIT), we predicted that right cathodal stimulation (left FHA) would lead to higher perceived success importance to get the reward determining higher effort in the unfixed task demand compared to the left cathodal stimulation and both stimulation conditions in the fixed task. As predicted, PEP reactivity was stronger in the unfixed condition following right cathodal stimulation compared to left cathodal stimulation and both stimulation conditions in the fixed task. Importantly, this effect was observed across both female and male participants, extending earlier neuromodulation findings (previously shown only in female samples) to both sexes. Overall, the results indicate that dlPFC neuromodulation can lead to higher effort by shifting frontal asymmetry and enhancing the perceived success importance in reward‐driven tasks.

## Abstract 2

This study advances our understanding of approach motivation and effort by demonstrating that frontal hemispheric asymmetry modulates HD‐tDCS effects on effort‐related cardiac responses across both male and female participants. Right cathodal stimulation over the dlPFC (inducing greater relative left frontal hemispheric asymmetry) increased pre‐ejection period reactivity compared to left cathodal stimulation, but only within an unfixed task demand context where participants could earn monetary rewards through self‐paced performance. In contrast, when task demand was fixed and easy (requiring only 50% accuracy to earn rewards), cardiovascular effort remained consistently low regardless of the stimulation condition. Critically, these neuromodulation effects were observed across both sexes, extending previous findings that were limited to female samples and establishing the generalizability of dlPFC stimulation effects on reward‐motivated effort.

## 1 Introduction

Much of our behavior is motivated by rewards—such as working diligently to earn a promotion or training hard to win a competition. Yet, sensitivity to rewards (SR) differs among individuals, and those with lower responsiveness often find it challenging to maintain motivation in their everyday lives (e.g., Brinkmann and Franzen 2013; Franzen and Brinkmann 2015). Encouragingly, recent studies suggest that motivation for anticipated rewards can be increased (Framorando, Delobel, and Razzetto 2025; Framorando, Gendolla, and Gable 2025). In two experiments, Framorando, Delobel, and Razzetto (2025) and Framorando, Gendolla, and Gable (2025) demonstrated that inducing greater relative left frontal hemispheric asymmetry (FHA)—which is associated with approach motivation (e.g., Harmon‐Jones and Gable 2018; Kelley et al. 2017)—enhanced SR and effort intensity in reward‐driven tasks compared to control conditions. However, these effects were observed exclusively in female samples, leaving open the question of whether they extend to males. To address this, the present study seeks to determine if this effect generalizes across both sexes.

### 1.1 Rewards and Effort

Motivational intensity theory, which is grounded in a resource conservation principle, proposes that effort invested in instrumental tasks aimed at achieving personal goals depends on perceived task difficulty and success importance (i.e., the importance of successfully performing the instrumental task) (Brehm and Self 1989).^1^ If task difficulty is clear and fixed, effort should rise with increasing difficulty until the effort required surpasses what is justified by the importance of success. When the required effort exceeds the maximally justified level, individuals should disengage to avoid wasting resources. In situations where task difficulty is unclear or unfixed (i.e., the performer can choose any difficulty level), difficulty cannot serve as an indicator of the necessary effort. Under these circumstances, individuals rely on success importance to minimize resource waste: the greater the success importance, the greater the effort invested. Although success importance does not allow individuals to precisely invest the exact effort required, it helps them avoid investing more than justified (Richter 2013).

Critically, anticipated rewards can determine success importance, subsequently influencing effort, particularly when success importance has a clear effect on effort (for instance, when task demand is unclear or unfixed). For example, in tasks of unclear difficulty, participants who could earn higher monetary incentives mobilized higher resources (e.g., Richter and Gendolla 2009). Importantly, individuals with higher SR should value positive consequences of rewards more strongly. Consequently, the higher the SR, the more pronounced the effect of rewards on success importance. Research on anhedonia supports this (e.g., Brinkmann and Franzen 2013; Franzen and Brinkmann 2015). Dysphoric participants—who experience low SR—demonstrated reduced effort in reward‐available tasks, regardless of reward magnitude. Additional research replicated these findings with social rewards and among participants diagnosed with major depressive disorder (e.g., Brinkmann et al. 2014; Franzen et al. 2019).

### 1.2 Approach Motivation, Sensitivity to Rewards and Effort

Recent research suggests that it is possible to enhance the impact of rewards on effort (Framorando, Delobel, and Razzetto 2025; Framorando, Gendolla, and Gable 2025). Approach motivation is grounded in a behavioral approach system (BAS) that responds to reward and non‐punishment signals (Gray 1970): increased BAS activation is associated with increased SR. Importantly, approach motivation has been consistently associated with differences in FHA (Dawson et al. 1992; Harmon‐Jones and Allen 1997, 1998; Harmon‐Jones et al. 2009; Sutton and Davidson 1997). Specifically, a large body of EEG research shows that greater left‐ than right‐frontal activity (greater relative left FHA) is linked to trait approach motivation. Frontal asymmetry is typically operationalized as the left–right difference in alpha‐band (8–13 Hz) power after log transformation, as alpha power inversely reflects cortical activity (e.g., Shagass 1972). For example, Harmon‐Jones and Allen (1998) showed that higher BAS scores were associated with greater relative left‐frontal activity. Supporting this, Schutter et al. (2008) combined EEG, BIS/BAS scales, and transcranial magnetic stimulation to demonstrate that greater relative left cortical excitability was associated with approach motivation. Other studies have manipulated FHA directly to alter motivational direction—using nostril breathing or unilateral facial or hand contractions—to induce approach state (e.g., Harmon‐Jones 2006; Schiff and Lamon 1989; Werntz et al. 1987). For instance, left‐hand contractions have been shown to elicit right FHA (Harmon‐Jones 2006).^2^

Importantly, noninvasive stimulation can also shift FHA to induce approach motivation: tDCS can alter cortical excitability (Nitsche and Paulus 2000), with HD‐tDCS's 4 × 1 ring enabling focal effects (Kuo et al. 2013; Parlikar et al. 2021). Placing the cathode (inhibitory stimulation) over the right dlPFC surrounded by anodes (excitatory stimulation) can reliably induce greater relative left FHA, increasing approach motivation and SR (e.g., Kelley et al. 2017; Parlikar et al. 2021).

Following this principle, in two recent studies, we applied HD‐tDCS to increase SR and effort in tasks involving monetary incentives (Framorando, Delobel, and Razzetto 2025; Framorando, Gendolla, and Gable 2025). Specifically, we tested whether HD‐tDCS–induced approach motivation would affect effort‐related cardiovascular responses. We hypothesized that right cathodal stimulation over the dlPFC—decreasing right frontal activity and thereby enhancing left frontal activity—would increase SR and, in turn, increase effort when task demands are unclear or unfixed, contexts in which SR typically affects effort. As predicted, induced greater relative left FHA (induced with right cathodal stimulation) led to stronger effort‐related cardiac responses—compared to greater relative right FHA (induced with left cathodal stimulation) and sham stimulation—under both unclear and unfixed task conditions. However, this effect appeared only in female participants: one study showed a contrasting pattern in males, and the other included only females, leaving generalization to males unresolved (Framorando, Delobel, and Razzetto 2025; Framorando, Gendolla, and Gable 2025).

This is particularly relevant as it aligns with growing evidence that tDCS effects, particularly over the prefrontal cortex, can differ by biological sex (e.g., Gao et al. 2018; Lapenta et al. 2012; Palmisano et al. 2021; Weller et al. 2023; Yang et al. 2018). For instance, Gao et al. (2018) reported sex‐differentiated responses to dlPFC tDCS in a “cheap talk” sender–receiver paradigm, with right‐anodal/left‐cathodal stimulation reducing deceptive behavior in women but not in men. Similarly, Weller et al. (2023) found that men and women responded differently to tDCS combined with cognitive training, with women showing larger performance gains on a demanding arithmetic task. Martin et al. (2017) further found that biological sex moderated HD‐tDCS effects on the Reading the Mind in the Eyes Test (Baron‐Cohen et al. 2001), with improved outcomes for women after anodal stimulation to dmPFC. Together, these findings suggest that women may be more sensitive to neuromodulation with tDCS, which could account for the female‐specific effects observed in our past work. Accordingly, we test whether the effects observed in female participants generalize to male participants or remain female‐specific.

### 1.3 Measuring Effort

Drawing on Obrist's work (Obrist 1981), which demonstrated that the sympathetic nervous system is activated when individuals engage in *active coping* tasks—situations in which successful performance allows for outcome control—Wright (1996) proposed that increases in sympathetic myocardial activity in such contexts reflect higher effort investment. Beta‐adrenergic sympathetic activity impacts cardiac contractile force, which is especially mirrored by the pre‐ejection period (PEP)—the time interval between the onset of left ventricular depolarization and the opening of the left aortic valve (Berntson et al. 2024). Since cardiac contractility contributes to cardiac output, some researchers have used systolic blood pressure (SBP) to estimate effort (e.g., Gendolla et al. 2012, 2019; Richter et al. 2016). However, SBP is also affected by peripheral vascular resistance (Levick 2003). Other studies have used heart rate (HR) as an effort indicator (e.g., Rogers 1969). However, HR is also affected by parasympathetic influences. Consequently, changes in PEP remain the most valid and sensitive index of effort (Kelsey 2012), as consistently demonstrated in prior work (e.g., Chatelain and Gendolla 2015; Framorando and Gendolla 2018, 2019a; Framorando et al. 2023, 2024a; Lasauskaite Schüpbach et al. 2014; Freydefont et al. 2012).

Critically, PEP should be interpreted alongside HR and diastolic blood pressure (DBP) to account for potential preload (ventricular filling) and afterload (arterial pressure) influences on myocardial contractility. Decreases in PEP should be attributed to beta‐adrenergic sympathetic activation only when they are not accompanied by parallel reductions in HR or blood pressure (Sherwood et al. 1990).

### 1.4 The Present Study

We examined the effects of HD‐tDCS targeting the dlPFC on effort‐related cardiovascular responses during a mental task with fixed and clear versus unfixed difficulty. In both task conditions, participants had the opportunity to earn money based on performance. Drawing on principles observed in TMS research, where right hemisphere inhibition enhances left hemisphere activity, we induced greater relative left FHA by applying cathodal HD‐tDCS over the right dlPFC (Right Cathodal Stimulation—RCS). Conversely, greater relative right FHA was induced via cathodal HD‐tDCS over the left dlPFC (Left Cathodal Stimulation—LCS).

We predicted that RCS would enhance approach motivation and increase SR relative to LCS through greater relative left FHA. In the presence of moderate monetary incentives, the enhanced SR should increase success importance and lead to increased effort in the unfixed task. In contrast, we expected that LCS would lead to greater relative right FHA. This should produce lower SR and success importance compared to RCS, resulting in lower effort in the same task. For the fixed and easy task condition, success importance should have little impact on effort. According to MIT (Brehm and Self 1989), effort in such tasks is primarily determined by low perceived task demand. Therefore, no neuromodulation effects were expected under these conditions.

## 2 Materials and Methods

### 2.1 Participants

89 right‐handed participants (44 men) from various faculties at the University of Geneva took part in the present study to align with previous studies on brain stimulation and effort‐related cardiovascular responses (Framorando, Delobel, and Razzetto 2025; Framorando, Gendolla, and Gable 2025). This sample size was based on a previous experiment (Framorando, Delobel, and Razzetto 2025) which showed that a sample around 25 participants per cell should be sufficient to obtain strong evidence (BF > 10) for our hypotheses (Van Doorn et al. 2021). We computed a sensitivity analysis by varying population‐level expected effects (class = “b”) effect {0.5, 1.0, 1.5, 2.0, 2.5}, which all showed BFs > 10. Participants received CHF 30 (approximately 33.5 USD) as compensation for their time and participation. After an initial check of signal quality and outliers, two participants were excluded due to technical issues during tDCS administration. This resulted in a final sample of *N* = 87 with a mean age of 23.23 years (SE = 0.42; median = 22.00; range = 18–36).

### 2.2 Design

Each participant was randomly placed into one of two stimulation conditions: left cathodal or right cathodal. Approximately half of the participants first completed a task of fixed and easy difficulty, followed by a task of unfixed difficulty. The remaining participants completed the tasks in the opposite order. This produced a mixed design, with stimulation condition (left vs. right cathodal) as a between‐subject factor and task demand type (fixed and easy vs. unfixed) as a within‐subject factor.

### 2.3 Materials and Apparatus

#### 2.3.1 Physiological Measures

PEP and HR were recorded using a Cardioscreen 1000 system (Medis, Ilmenau, Germany), both derived from synchronized ECG and ICG signals. Two pairs of Ag/AgCl electrodes (Medis) were positioned on the left side of the participants' neck and chest (at the left mid‐axillary line aligned with the xiphoid process). The signals were amplified and digitized at 1000 Hz, then processed offline with a 50 Hz low‐pass filter using BlueBox 2. V1.22 software (Richter 2010). Automatic R‐peak detection was performed using a threshold‐based algorithm, followed by visual verification. The first derivative of the thoracic impedance change was calculated, producing the dZ/dt signal, which was averaged over a 5‐min period based on detected R‐peaks. The B point was identified through the RZ interval from valid cardiac cycles (Lozano et al. 2007), with all detections visually reviewed and manually corrected when necessary, following Sherwood et al.'s (1990) protocols. Heart rate was derived from the intervals between heartbeats obtained via the Cardioscreen system.

Systolic and diastolic blood pressure were measured at 1‐min intervals using a Dinamap ProCare monitor (GE Healthcare, Milwaukee, WI, USA) and averaged to obtain a single value for the last 5 min of the baseline period and for the entire task period (5 min). An automatic blood pressure cuff was placed on the non‐dominant arm, just above the elbow, and inflated automatically at each measurement interval.

#### 2.3.2 Application of HD‐tDCS

Brain stimulation was delivered using a Neuroelectric StarStim 8‐channel device (Barcelona, Spain). The target electrode locations were determined using a 10–20 EEG system (Jasper 1958). To induce left cathodal stimulation (LCS), the cathode was placed over F3; the anodes over AF3, F1, F5, and FC3. To induce RCS, the cathode was positioned over F4 and the anodes over AF4, F2, F6, and FC4. HD‐tDCS was administered at an intensity of 2 mA for 21 min, including 30 s ramp‐on and 30 s ramp‐off times. No sham stimulation was included in this study.

#### 2.3.3 Cognitive Tasks

The first task was a modified version of a d2 attention task (Brickenkamp 1981). Participants were instructed to determine whether the stimulus was a “d” accompanied by exactly two apostrophes, positioned above, below, or both above and below the target letter. Responses were given by pressing “yes” or “no” keys with two fingers of their dominant hand. Incorrect trials included the letter “d” or “p” with 0, 1, 3, or 4 apostrophes or the letter “p” with exactly 2 apostrophes. The second task followed the same structure as task 1 except that participants had to identify the letter “b” with exactly 2 asterisks. Incorrect trials were composed of the letters “b” or “q” combined with 0, 1, 3, or 4 asterisks or the letter “q” with 2 asterisks.

#### 2.3.4 Task Manipulation

##### 2.3.4.1 Fixed and Easy Task Demand

**FIGURE 1:**
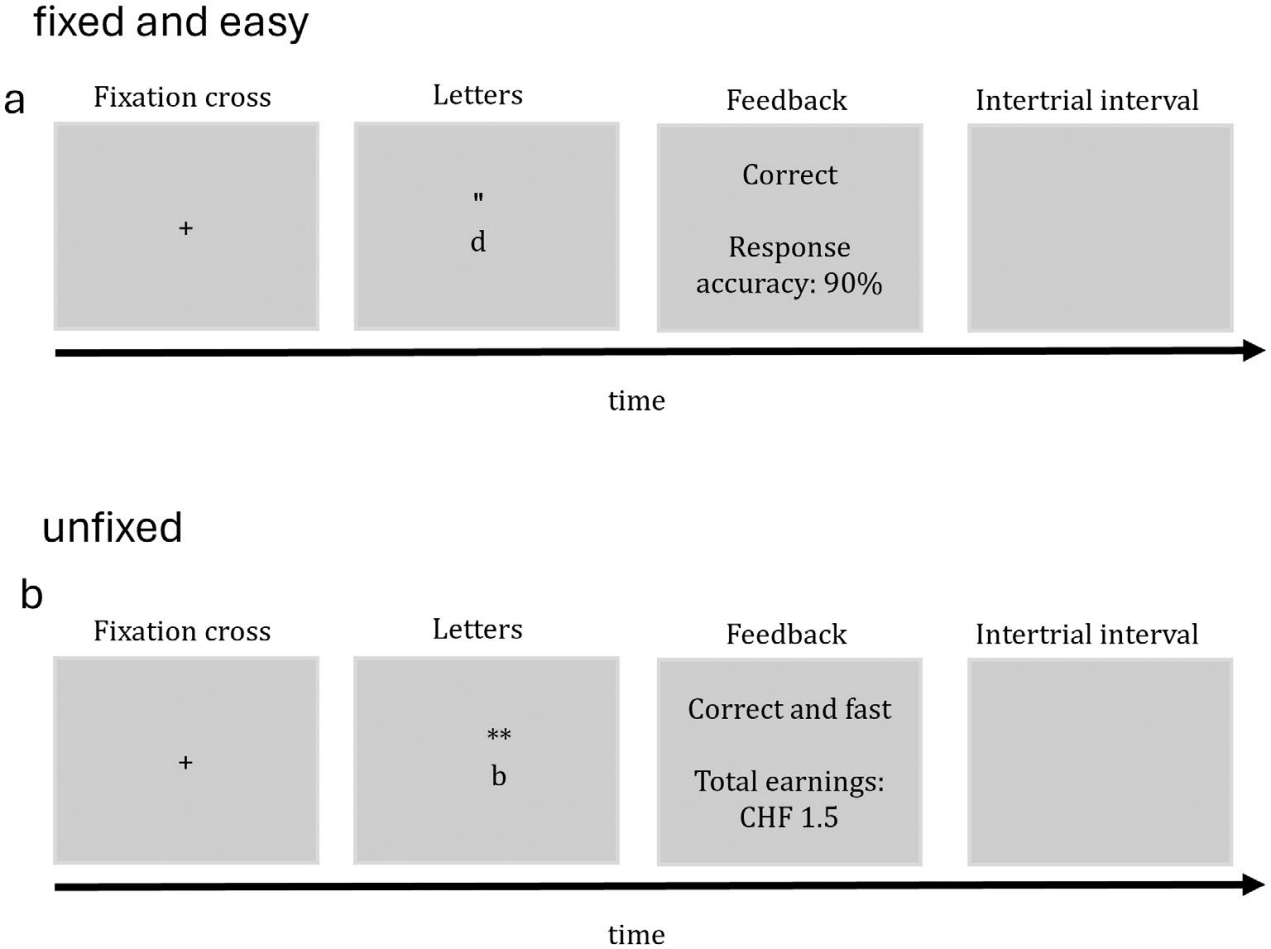
Example of an experimental trial. (a) Task 1 under the fixed and easy condition, while (b) task 2 under the unfixed condition. Note that for half of the participants, contrary to what is depicted in Figure 1, the unfixed task demand appeared in task 1 while the fixed and easy task demand appeared in task 2 (counterbalanced order). In (a), the target stimulus is the letter “d” with exactly two apostrophes. Participants should press the green keyboard key to respond “yes.” Similarly, in (b), the letter “b” is accompanied by exactly two asterisks. Participants should respond “yes” using the green keyboard key.

In this condition, participants were told they would receive 7.5 Swiss Francs (8.7 USD) if their performance exceeded 50% of correct responses. Each trial began with a fixation cross displayed for 1000 ms, followed by a target letter for 3500 ms. If no response was given within this timeframe, a feedback message (“Please answer faster”) was shown for 3500 ms along with the current success rate (the trial was recorded as incorrect). If participants responded within the allotted time, they received feedback indicating whether the response was correct or incorrect—depending on the accuracy of their response—along with the updated success rate. The trial ended with a blank screen lasting between 2 and 5 s. An example of the experimental trial for the fixed and easy task demand condition is depicted in Figure 1a. The entire task included 25 trials and lasted 5 min.

##### 2.3.4.2 Unfixed Task Demand

In this condition, participants were informed that they could earn up to 7.5 Swiss Francs (8.7 USD) in total. Each trial started with a fixation cross displayed for 1000 ms, followed by the letter which remained onscreen until a response was made. Monetary rewards were contingent on both speed and accuracy: fast and correct responses (< 484 ms) were rewarded with 0.30 Swiss Francs (USD 0.36) per trial, slow but correct responses (> 484 ms) yielded 0.10 Swiss Francs (USD 0.12), and incorrect responses yielded no reward (0.00 Swiss Francs). After each response, feedback was presented for 3500 ms, indicating whether they were fast and correct, slow and correct, or incorrect along with their total accumulated earnings. Each trial concluded with a blank screen lasting between 2 and 5 s. An example of the experimental trial for the unfixed task demand condition is depicted in Figure 1b. The task ended after 5 min.

### 2.4 Procedure

All the procedures and measurements were approved by the local ethics committee prior to the start of data collection. To minimize potential experimenter bias (e.g., Gilder and Heerey 2018), the experimenter was unaware of the study's hypotheses and experimental conditions. Before the experimental sessions began, participants completed a safety questionnaire to identify any contraindications such as the presence of a pacemaker, medical implants, or a history of brain damage. They were also asked to read and sign a written informed consent form, which outlined the potential risks of tDCS and detailed the experimental procedures. Additionally, participants completed the BIS/BAS scale (Carver and White 1994). Individuals with epilepsy, frequent headaches, or metallic implants in the head were excluded for safety reasons.

Upon arrival at the lab, participants were greeted and invited to sit comfortably in front of a computer. The experimenter launched the computer program with the experimental protocol (Nuxt.js; Version 3.12.4, NuxtLabs, San Francisco, CA, USA). Before beginning the experiment, tDCS and physiological sensors were applied to each participant.

Participants first reported their age and biological sex. Next, they watched a neutral space‐theme documentary lasting approximately 20 min while tDCS was administered. This was followed by a second neutral documentary, this time focused on trees (7 min), during which cardiovascular baseline activity was recorded. Participants were instructed to remain relaxed and refrain from moving during this period. After the initial baseline period, participants completed the first version of the d2 attention task, while physiological recordings continued. This was followed by a short baseline period of a 3‐min documentary about lemurs, in order to restore physiological baseline. No physiological data were collected during this baseline. Following this, participants completed a second version of the d2 attention task where physiological data were recorded (see Section 2.3.3). After the completion of both attention tasks, participants provided information on their native language, level of French proficiency, cardiovascular health status, and any medication use. The session concluded with a debriefing phase, during which participants were informed about their individual performance and whether they succeeded in the tasks. They were also asked to guess the aim of the study and to report any discomfort or pain experienced during the HD‐tDCS procedure.

### 2.5 Data Analysis

The dataset and associated coding script used in this study are hosted on the Geneva Motivation Lab server: https://amb3.genevamotivationlab.ch. The data analysis process was preregistered on the Aspredicted platform (https://aspredicted.org/hwbd‐67x4.pdf). Statistical analyses were conducted using RStudio (R Core Team 2023) and JASP (JASP Team 2023) software.

To assess the impact of HD‐tDCS on cardiovascular responses, we employed Bayes factors to compare our hypothesized effects against the null hypothesis (Masson 2011). The choice of Bayesian approach was made to ensure continuity with the analytical approach used in Framorando, Delobel, and Razzetto (2025) and Framorando, Gendolla, and Gable (2025) and because Bayesian analysis enables a graded interpretation of results, quantifying support for either the null or alternative hypothesis (Dienes 2014; Rouder et al. 2009). Our main hypothesis posited that right cathodal stimulation (RCS) would lead to higher effort‐related cardiovascular reactivity compared to left cathodal stimulation (LCS) in the unfixed task demand condition, and compared to both stimulation conditions in the fixed and easy task demand. To test this hypothesis, we implemented an a priori contrast with predefined weights: −3 was assigned to the RCS condition in the unfixed task demand, and +1 for LCS under the same task condition, and for both stimulation conditions in the fixed and easy task demands.

For variables for which we had no a priori contrast expectation, we computed 2 (Stimulation) × 2 (Task) Bayesian ANOVA.^3^

## 3 Results

### 3.1 BIS/BAS Scores

To check for potential dispositional differences in approach/avoidance motivation, preliminary one‐way Bayesian ANOVAs were conducted with HD‐tDCS condition as the factor for BIS, reward responsiveness, fun‐seeking, and drive scores. The resulting Bayes factors and posterior probabilities were low for BIS, reward responsiveness, fun‐seeking, and drive (BFs ≤ 0.339; *p*(*M*|data) < 0.253). These findings show low support for condition‐based differences in BIS/BAS scores at baseline.

### 3.2 Cardiovascular Baseline

Cardiovascular baseline scores were computed over the last 5 min of the habituation phase.^4^ One‐way Bayesian ANOVAs of the baseline scores were computed for each cardiovascular index to examine any baseline differences between left and right stimulation conditions. The analysis yielded low Bayes factors and posterior probabilities (BFs < 0.373; *p*(*M*|data) < 0.272), indicating minimal evidence for condition‐based differences at baseline. Mean and standard error values for each stimulation condition at baseline are provided in Table 1.

**TABLE 1:** Baseline mean and standard error (in parenthesis) of pre‐ejection period, blood pressure, and heart rate.

Cardiac index	LCS	RCS
PEP	104.65 (1.81)	102.32 (1.97)
SBP	104.63 (2.24)	103.54 (1.40)
HR	71.33 (1.98)	73.95 (1.39)
DBP	60.14 (1.18)	61.34 (1.18)

*Note:* DBP = diastolic blood pressure (in mmHg), HR = heart rate (in beats/min), LCS = Left Cathodal Stimulation, PEP = pre‐ejection period (in ms), RCS = Right Cathodal Stimulation, SBP = systolic blood pressure (in mmHg).

### 3.3 Cardiovascular Reactivity

Cardiovascular reactivity was computed by subtracting each participant's baseline value from the values of PEP, SBP, HR, and DBP assessed during the tasks (fixed and easy and unfixed).^5^ For each index, we tested for the potential effect of task order (i.e., whether participants began with the fixed and easy task or the unfixed task). To this end, we conducted 2 (stimulation: left cathodal vs. right cathodal) × 2 (task: fixed and easy vs. unfixed) Bayesian ANOVAs, including task order as an additional factor. For all cardiovascular parameters, models including task order yielded low Bayes Factors and posterior probabilities (BFs < 1.709, *p*(*M*|data) < 0.125). Consequently, order was not included in the subsequent analyses.

To examine the potential effects of sex, we conducted preliminary 2 (stimulation: left cathodal vs. right cathodal) × 2 (task: fixed and easy vs. unfixed) × 2 (sex: female, male) Bayesian ANOVAs. For all cardiovascular parameters, models including sex yielded low Bayes Factors and posterior probabilities (BFs < 1.236, *p*(*M*|data) < 0.143). Consequently, sex was not included in the subsequent analyses.

#### 3.3.1 PEP Reactivity

**FIGURE 2:**
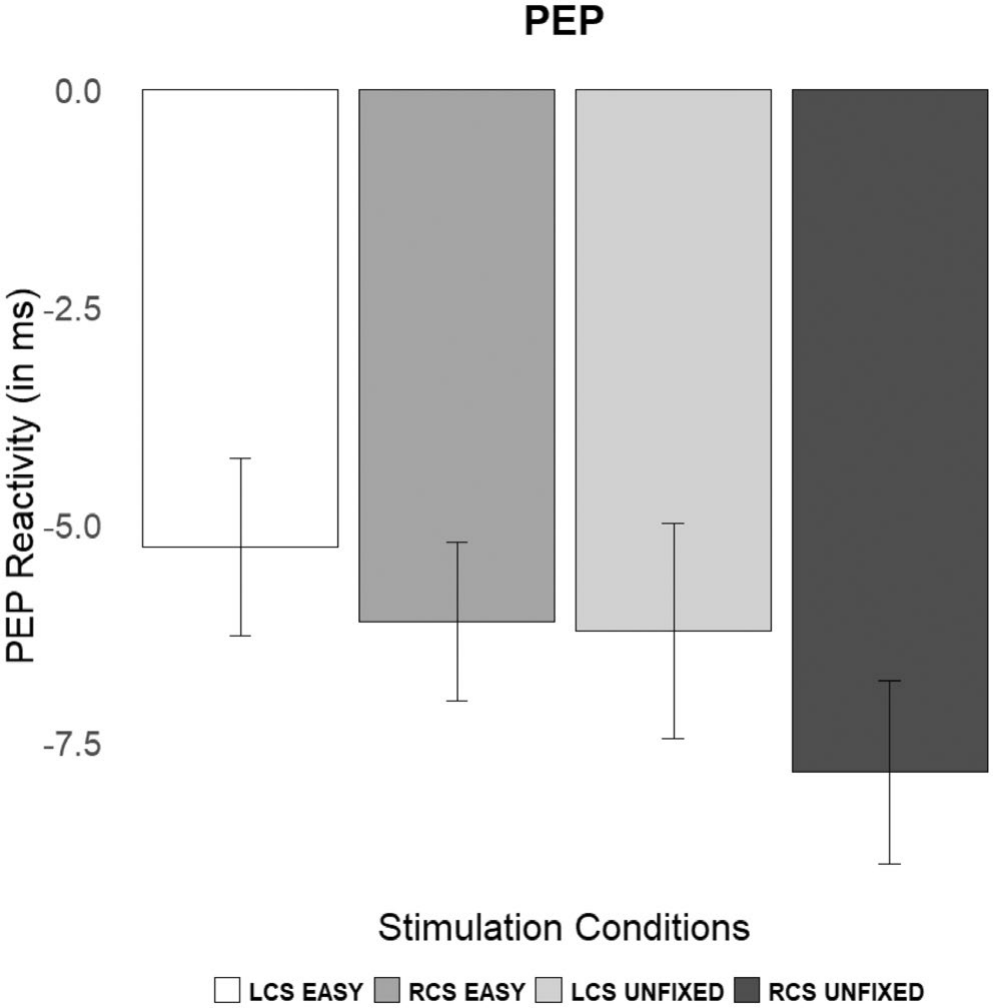
Cell means (±1 standard error) of pre‐ejection period (in ms) reactivity in the experimental conditions. LCS = Left Cathodal Stimulation, RCS = Right Cathodal Stimulation.

Supporting our hypothesis, the directional Bayesian 3:1 contrast yielded strong evidence for the model (BF = 18.417, *p*(*M*|data) = 0.949). As expected, and illustrated in Figure 2, PEP reactivity for the unfixed task demand was stronger following right cathodal stimulation (*M* = −7.84, SE = 1.05) compared to left cathodal (*M* = −6.22, SE = 1.24) and compared to both stimulation conditions during the fixed and easy task demand stimulation conditions (left cathodal: *M* = −5.26, SE = 1.02; right cathodal: *M* = −6.11, SE = 0.91).^6^ The corresponding Bayesian standardized effect size indicated a large difference (posterior *d* = 1.35).

Furthermore, separate Bayesian analyses for the female and male subsamples provided moderate to strong evidence in support of the model for both groups (female sample: BF = 5.163, *p*(*M*|data) = 0.843; male sample: BF = 8.324, *p*(*M*|data) = 0.892). PEP reactivity in the unfixed task was stronger following RCS (female sample: *M* = −7.50, SE = 1.38; male sample: *M* = −8.20, SE = 1.63) compared to LCS (female sample: *M* = −5.62, SE = 1.41; male sample: *M* = −6.82, SE = 2.07) and compared to both LCS (female sample: *M* = −5.02, SE = 1.44; male sample: *M* = −5.50, SE = 1.47) and RCS (female sample: *M* = −6.56, SE = 1.11; male sample: *M* = −5.63, SE = 1.48) conditions when task demand was fixed and easy.

#### 3.3.2 SBP, DBP and HR Reactivity^7^

The Stimulation × Task interaction for SBP, HR and DBP cardiovascular reactivity reveals low support for the tested models (BFs < 0.417; *p*(*M*|data) < 0.294).

Cardiovascular reactivity means and standard errors are presented in Table 2.

**TABLE 2:** Cardiovascular reactivity scores and standard error (in parenthesis) of blood pressure and heart rate.

Cardiac index	LCS easy	RCS easy	LCS unfixed	RCS unfixed
SBP	6.53 (0.99)	8.61 (1.12)	7.72 (1.17)	9.62 (1.28)
HR	2.82 (0.64)	3.25 (0.62)	2.92 (0.67)	3.28 (0.68)
DBP	3.35 (0.59)	5.39 (0.62)	3.69 (0.64)	5.66 (0.80)

*Note:* DBP = diastolic blood pressure (in mmHg), HR = heart rate (in beats/min), LCS = Left Cathodal Stimulation, RCS = Right Cathodal Stimulation, SBP = systolic blood pressure (in mmHg).

### 3.4 Task Performance

Bayesian ANOVAs for response accuracy (percentage of correct responses across the total amount of trials) and reaction times showed that the best models support only the main effect of task (BFs > 77.179, *p*(*M*|data) > 0.678). Response accuracy and reaction times were higher and slower in the fixed and easy task (response accuracy: *M* = 98.58, SE = 0.49|reaction times: *M* = 813.96; SE = 25.55) compared to the unfixed task (response accuracy: *M* = 90.41; SE = 0.94|reaction times: *M* = 630.30; SE = 50.74). Models including stimulation and stimulation × task interaction revealed lower Bayes factors and posterior distributions fit (BFs < 0.366, *p*(*M*|data) < 0.242). This provides little evidence that the stimulation or its interaction with task affected task performance.

### 3.5 Funnel Debriefing

No participant correctly guessed the purpose of the experiment during the funnel debriefing. Furthermore, no participant reported pain associated with the experimental HD‐tDCS manipulation in any stimulation condition.

## 4 Discussion

The present study provides the first mixed‐sex evidence that modulating FHA with HD‐tDCS affects effort‐related cardiac reactivity in a context‐dependent manner in both female and male samples. Consistent with MIT (Brehm and Self 1989) and prior sex‐limited findings (Framorando, Delobel, and Razzetto 2025; Framorando, Gendolla, and Gable 2025), RCS over dlPFC—which enhances relative left FHA—increased PEP reactivity when task difficulty was unfixed compared to contralateral stimulation, whereas PEP reactivity remained low following both left and right stimulations when task demand was fixed and easy. As hypothesized, RCS enhanced left frontal hemispheric asymmetry, which enhanced approach motivation and SR—compared to LCS (Coan and Allen 2003; Harmon‐Jones and Gable 2018; Kelley et al. 2017). In the presence of moderate monetary incentives, this elevated SR increased the success importance. According to MIT, when task demands are unfixed, perceived success importance becomes the primary determinant of effort (Brehm and Self 1989; see Gendolla et al. 2019; Richter et al. 2016 for recent reviews). By contrast, when task difficulty is fixed, effort intensity is primarily determined by perceived task demand, with success importance defining the upper limit at which individuals disengage and stop investing effort. Individuals with lower success importance are expected to disengage at lower difficulty levels than those with high success importance, as the maximum effort they are willing to invest to achieve success is smaller. However, when task demand is low, the required effort is minimal and therefore easily justified—so manipulations of success importance should not affect effort. Consequently, under a fixed and easy task demand, effort intensity should remain low. Thus, the increased success importance following RCS resulted in higher effort specifically in the unfixed task demand condition, with no neuromodulation effects observed in the fixed and easy condition as predicted.

These findings are in line with previous approach‐avoidance work (Fecteau et al. 2007; Framorando, Delobel, and Razzetto 2025; Framorando, Gendolla, and Gable 2025; Knoch, Gianotti, et al. 2006; Knoch, Pascual‐Leone, et al. 2006; Ohmann et al. 2018). Knoch, Gianotti, et al. (2006) showed that inhibitory TMS to the right dlPFC—thereby increasing relative left FHA—led participants to prefer riskier, higher‐reward choices over safer, lower‐reward ones. Likewise, Ohmann et al. (2018) found that left anodal stimulation (LAS) of the dlPFC (also reflecting greater relative left FHA) made participants favor harder trials with larger payoffs. Consistent effects emerged in our lab's earlier effort study (Framorando, Delobel, and Razzetto 2025; Framorando, Gendolla, and Gable 2025). In Framorando, Gendolla, and Gable (2025), under an unclear task demand with moderate incentives, female participants receiving RCS mobilized more resources than those with left cathodal or sham. This is because right cathodal stimulation heightened left FHA, increasing SR and perceived success importance in the presence of moderate incentives, which directly determine effort in such a task context. Altogether, neuromodulation that promotes left FHA appears to intensify approach motivation and SR, affecting decision making and physiological effort.

A key goal was to clarify earlier ambiguities regarding sex differences. Prior studies either found effects only in female samples (Framorando, Gendolla, and Gable 2025) or included female participants only (Framorando, Delobel, and Razzetto 2025). Because accumulating evidence shows that tDCS effects can vary by sex in other cognitive domains (Gao et al. 2018; Weller et al. 2023), we could not assume that the neuromodulation–effort link would be identical for male and female participants. Importantly, we found the expected pattern in both male and female samples, extending approach/avoidance‐effort research on male samples (Framorando, Delobel, and Razzetto 2025; Framorando, Gendolla, and Gable 2025).

In this study, we specifically anticipated an effect on PEP, with weaker or no effects on other cardiovascular measures, based on the findings of Framorando, Delobel, and Razzetto (2025), which the present work directly replicates. We tested other cardiovascular indexes using mixed Bayesian ANOVA analyses instead of directional contrasts. As predicted, the results confirmed the PEP effect and showed no clear patterns for the other measures—consistent with Framorando, Delobel, and Razzetto (2025). Although mixed Bayesian ANOVA analyses may be less sensitive to subtle directional contrast effects, we also explored specific directional contrasts for theoretical completeness. These contrasts provided evidence for corresponding effects on SBP and DBP, but not on HR. While these additional tests were not part of our original analysis plan, the pattern is theoretically coherent: SBP—and to a lesser extent DBP—are influenced by cardiac output, which reflects cardiac contractile force. Previous studies have likewise reported corresponding effects on PEP, SBP, and sometimes DBP (e.g., Brinkmann et al. 2009; Falk et al. 2022; Framorando and Gendolla 2019a; Framorando et al. 2024a; Mazeres et al. 2019; Richter et al. 2008). Importantly, in the present study, PEP reactivity was not accompanied by concurrent decreases in blood pressure or HR. Therefore, the observed PEP responses can be attributed to beta‐adrenergic sympathetic nervous system activation rather than changes in cardiac preload or vascular afterload (see Sherwood et al. 1990).

In contrast to the effects observed on PEP reactivity, the manipulation did not affect task performance measures. However, we did not expect that variations in effort would be automatically associated with variations in performance. While some studies did report parallel changes in cardiovascular effort indices and performance (Framorando and Gendolla 2018; Gendolla and Silvestrini 2010), others did not (Framorando and Gendolla 2019a, 2019b; Framorando et al. 2021; Lasauskaite Schüpbach et al. 2014; Wang et al. 2023). The link between effort and performance is more complex than simply linear. It is difficult to predict performance (behavioral output) from effort intensity (behavioral input), as performance depends besides effort also, or even more so, on task‐related ability, persistence, and the applied strategies (Locke and Latham 1990). Thus, individual differences in capacity and strategy use can moderate whether increased effort yields measurable improvements in accuracy or RT, so robust PEP reactivity alongside unchanged performance is not surprising and does not undermine the effort interpretation.

### 4.1 Limitations

A potential limitation is the absence of concurrent sham stimulation. Although a previous study within the same research program included a sham condition and showed that right‐cathodal HD‐tDCS over the dlPFC increased effort‐related cardiac responses compared to both left‐cathodal and sham control stimulation under unclear task demand (Framorando, Gendolla, and Gable 2025), the present design did not include a sham condition. This should be kept in mind when interpreting the results. Future studies could benefit from including both active and sham stimulations within the same experiment to further control for possible expectancy or other nonspecific influences.

Another limitation is the relatively homogeneous sample of right‐handed young university students, which constrains generalizability. We did not systematically assess additional participant characteristics or potential biological variations (e.g., hormonal status) that might affect responses. Random assignments should help balance unmeasured factors across conditions, but residual confounding cannot be ruled out. Future studies should record these variables (e.g., cycle phase, contraceptive status) and, where feasible, model them as moderators.

In the present study, SBP and DBP data for the first 26 participants were irretrievably lost due to a technical error following a software update. Although random assignment makes condition‐related missingness unlikely, the reduced sample size for these measures may have limited the statistical power of the mixed ANOVA to detect smaller effects, even though effects emerged in exploratory contrast analyses.

## 5 Conclusions

In summary, the present study contributes to research on neuromodulation and effort by showing that right cathodal stimulation led to higher effort‐related cardiovascular responses compared to left cathodal stimulation under conditions of an unfixed task demand. This effect is likely attributable to increased SR affecting effort levels in contexts involving monetary incentives, aligning with previous research on reward‐related effort dynamics (e.g., Franzen and Brinkmann 2015). In contrast, when task demands were fixed and easy, effort‐related cardiovascular responses remained low regardless of the hemispheric site of cathodal stimulation, consistent with the notion that under such conditions, the importance of success—and thus SR—has no effect on effort. Importantly, the stimulation effect on effort was observed consistently in both female and male participants, providing evidence against a sex‐specific influence of tDCS on effort.

## Author Contributions

**David Framorando:** conceptualization, funding acquisition, writing – original draft, methodology, writing – review and editing, software, formal analysis, data curation, project administration. **Andréa Razzetto:** investigation, writing – review and editing, project administration, data curation.

## Funding

This research was supported by an Ambizione grant from the Swiss National Science Foundation (SNF PZ00P1_216471), awarded to Dr. David Framorando.

## Conflicts of Interest

The authors declare no conflicts of interest.

## Data Availability

The data and data coding for the reported studies are available on the server from the GenevaMotivationLab: https://amb3.genevamotivationlab.ch/.

## Endnotes

^1^
Throughout, “task difficulty” and “success importance” refer to participants' perceptions of these constructs, consistent with motivational intensity theory (Brehm and Self 1989). Unless otherwise specified, all mentions of “task difficulty” and “success importance” denote these perceived levels.

^2^
Although previous research has proposed a link between anodal/cathodal stimulation and changes in frontal hemispheric asymmetry, this relationship has not yet been directly tested.

^3^
Since the present study is a direct replication of the original experiment with sex included as an additional factor, and no contrast effects were observed for SBP, DBP, and HR reactivity in Framorando, Delobel, and Razzetto (2025), we had no a priori expectation to find contrast effects on these variables. Accordingly, SBP, DBP, and HR were analyzed using a 2 (Stimulation) × 2 (Task) Bayesian ANOVA.

^4^
As detailed in the *Physiological Measures* section, for PEP, the dZ/dt signal was averaged over a 5‐min period based on detected R‐peaks, rather than the more traditional 1‐min intervals (e.g., Framorando et al. 2023, 2024a, 2024b). This longer averaging window was chosen to increase the number of cardiac cycles contributing to the average, thereby improving the reliability of B‐point detection, and to maintain consistency with the analyses reported in Framorando, Delobel, and Razzetto (2025), which the current study aimed to replicate.

^5^
PEP was calculated by averaging the dZ/dt signal over a 5‐min period based on detected R‐peaks, rather than the traditionally used 1‐min intervals (e.g., Framorando et al. 2023, 2024a, 2024b; Framorando, Delobel, and Razzetto 2025). This extended averaging window was selected to increase the number of cardiac cycles included, thereby improving the reliability of B‐point detection. Task scores for SBP, HR, and DBP were obtained by averaging the 1‐min measurements recorded throughout the tasks to obtain a single value for each task period, as with PEP.

^6^
In the unfixed task demand condition, the task did not impose a fixed number of motor responses. As a result, response frequency could vary across participants, raising the question of whether this variability might account for differences in cardiovascular reactivity. To examine this possibility, we conducted 2 (stimulation: left cathodal vs. right cathodal) × 2 (task: fixed and easy vs. unfixed) Bayesian ANOVAs including the number of motor responses as a covariate. Regarding the effect of the number of motor responses, the analysis showed low Bayes Factors (BFs < 0.495) and low posterior model probabilities (*p*(*M*|data) < 0.088) for PEP, SBP, and HR. Regarding DBP, the model including the main effect of tDCS and the number of motor responses yielded a moderate Bayes factor (BF = 8.131) and posterior model probabilities (*p*(*M*|data) < 0.462). However, analysis for specific effects showed low evidence for the number of motor responses (BFincl = 2.83). This suggests that variation in motor responses is unlikely to explain potential cardiovascular reactivity patterns.

^7^
For interested readers, exploratory a priori directional contrasts revealed that SBP and DBP reactivity patterns were supported by the Bayesian analyses, showing substantial evidence for the predicted direction (BFs > 13.963, *p*(*M*|data) > 0.933; means presented in Table 2). In contrast, the evidence for the HR contrast was weak (BF = 1.726, *p*(*M*|data) = 0.636).
